# Noninvasive hippocampal blood−brain barrier opening in Alzheimer’s disease with focused ultrasound

**DOI:** 10.1073/pnas.2002571117

**Published:** 2020-04-13

**Authors:** Ali R. Rezai, Manish Ranjan, Pierre-François D’Haese, Marc W. Haut, Jeffrey Carpenter, Umer Najib, Rashi I. Mehta, J. Levi Chazen, Zion Zibly, Jennifer R. Yates, Sally L. Hodder, Michael Kaplitt

**Affiliations:** ^a^Rockefeller Neuroscience Institute, West Virginia University, Morgantown, WV 26505;; ^b^Department of Neurosurgery, West Virginia University, Morgantown, WV 26505;; ^c^Department of Electrical Engineering and Computer Science, Vanderbilt University, Nashville, TN 37212;; ^d^Department of Neurological Surgery, Vanderbilt University, Nashville, TN 37212;; ^e^Department of Behavioral Medicine and Psychiatry, West Virginia University, Morgantown, WV 26505;; ^f^Department of Neurology, West Virginia University, Morgantown, WV 26505;; ^g^Department of Neuroradiology, West Virginia University, Morgantown, WV 26505;; ^h^West Virginia Clinical and Translational Science Institute, West Virginia University, Morgantown, WV 26505;; ^i^Department of Radiology, Weill Cornell Medical College, New York, NY 10065;; ^j^Department of Neurosurgery, Sheba Medical Center, Ramat Gan 52621, Israel;; ^k^Department of Neurological Surgery, Weill Cornell Medical College, New York, NY 10065

**Keywords:** focused ultrasound, hippocampus, Alzheimer’s disease

## Abstract

The blood–brain barrier (BBB) presents a significant challenge for treating brain disorders. The hippocampus is a key target for novel therapeutics, playing an important role in Alzheimer’s disease (AD), epilepsy, and depression. Preclinical studies have shown that magnetic resonance (MR)-guided low-intensity focused ultrasound (FUS) can reversibly open the BBB and facilitate delivery of targeted brain therapeutics. We report initial clinical trial results evaluating the safety, feasibility, and reversibility of BBB opening with FUS treatment of the hippocampus and entorhinal cortex (EC) in patients with early AD. Six subjects tolerated a total of 17 FUS treatments with no adverse events and neither cognitive nor neurological worsening. Post-FUS contrast MRI revealed immediate and sizable hippocampal parenchymal enhancement indicating BBB opening, followed by BBB closure within 24 h. The average opening was 95% of the targeted FUS volume, which corresponds to 29% of the overall hippocampus volume. We demonstrate that FUS can safely, noninvasively, transiently, reproducibly, and focally mediate BBB opening in the hippocampus/EC in humans. This provides a unique translational opportunity to investigate therapeutic delivery in AD and other conditions.

The hippocampus is critical in the pathogenesis of Alzheimer’s disease (AD), epilepsy, and depression (1). Patients with these disorders have significant unmet needs, driving efforts to develop treatments to address hippocampal pathology and dysfunction. A major challenge in the translation of therapies for brain disorders is the presence of the blood–brain barrier (BBB). To overcome the BBB, pharmaceuticals and biological treatments require systemic dose escalation or invasive, risky procedures like transarterial diuretic infusion and direct intracranial infusion (2, 3). Magnetic resonance (MR)-guided focused ultrasound (FUS) utilizes a transducer helmet with multiple ultrasound sources converging energy of varying intensity and frequency to a precise focal point in the brain. High-intensity FUS is used for treatment of tremor, while low-intensity FUS is being explored for BBB opening (4, 5). Low-intensity ultrasound energy beams cause intravenously (i.v.) administered microbubbles to oscillate, resulting in acoustic cavitation and transient opening of tight junctions in capillaries and the BBB (Fig. 1 *A* and *B*). Animal studies have demonstrated safe and reversible BBB opening (4, 6, 7) as well as reduction of amyloid-beta plaque, neurogenesis, and improvement of memory. Additionally, this technology enables targeted noninvasive delivery of genetic vectors and cells (6, 8–10). A proof of concept study in five AD patients demonstrated safe and temporary opening of the BBB in the white matter of the superficial dorsolateral prefrontal cortex (11). We now report early safety and feasibility outcomes in AD patients from a multicenter FUS treatment study targeting a substantial portion of the deep and complex structures of the hippocampus and entorhinal cortex (EC).

**Fig. 1.**
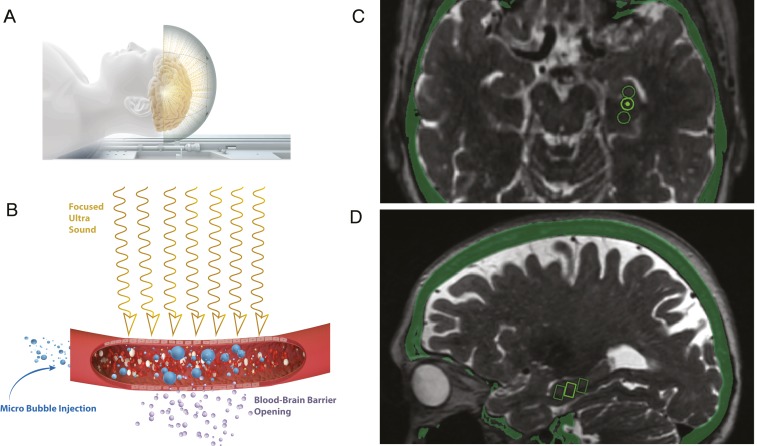
Illustration of FUS process and targeting. (*A*) The INSIGHTEC ultrasound system consists of a helmet with 1,024 ultrasound transducers attached to the MRI table. (*B*) Ultrasound beams travel transcranially to the target. Low-intensity (220 kHz) ultrasound energy beams interact with i.v. administered microbubbles. Subsequent oscillation of the microbubbles and acoustic cavitation cause transient opening of tight junctions in capillaries and open the BBB. Snapshot of the therapy planning software illustrating for a single subject (*C*) axial slice image of three targets in the hippocampus represented by circles, and (*D*) the estimated targeting volumes (5 × 5 × 7 mm^3^) represented as rectangles on the sagittal slice.

## Results

Six participants (five female and one male: ages 55 y to 73 y) with early AD were enrolled at the West Virginia University Rockefeller Neuroscience Institute (*n* = 4) and Weill Cornell Medical College (*n* = 2). FUS treatment with up to five targets of either the right (*n* = 2) or left (*n* = 4) hippocampus/EC was performed. A total of 17 treatment sessions have been completed.

**Fig. 2.**
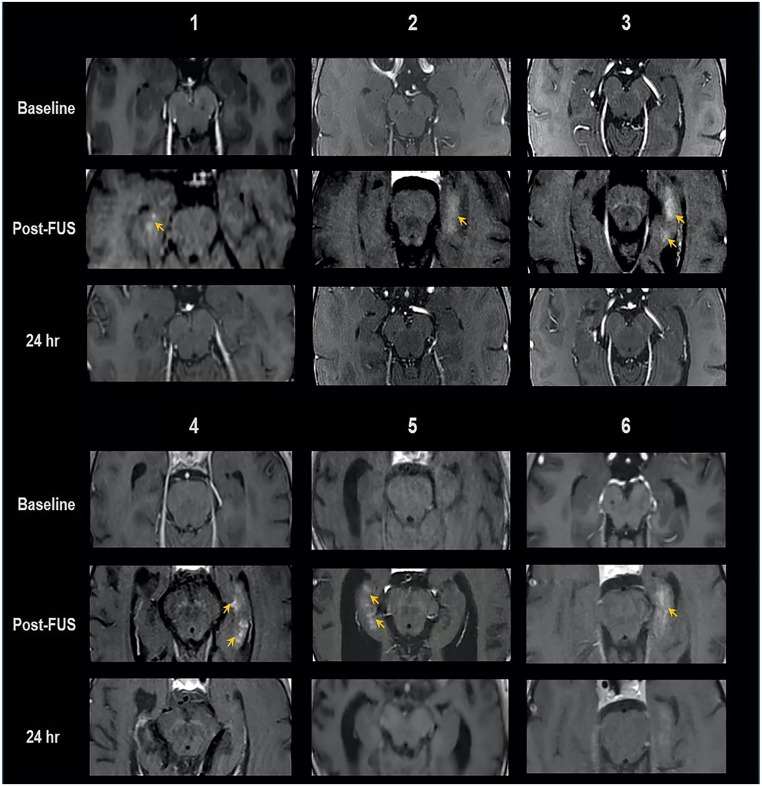
MRI evidence of BBB opening and closure in six subjects. Baseline, immediately post-FUS, and 24-h post-FUS contrast-enhanced T1 axial MRI shows parenchymal contrast enhancement at targeted sites (arrows), indicating BBB opening, and resolution at 24 h, following repeat contrast administration, indicative of BBB closure.

All participants tolerated the FUS procedure well and were discharged home within 24 h. There were no treatment-related adverse effects or neurological changes (up to 15 mo post-FUS). Formal cognitive assessments at 30 d after the last treatment in the first five subjects showed no clinically meaningful changes (*SI Appendix*). T2* MRI following FUS treatment and at subsequent follow-up did not indicate overt hemorrhage. MRI with gadobutrol IV contrast post-FUS treatment revealed immediate hippocampal parenchymal enhancement at the target region in all 17 treatment sessions, indicating enhanced BBB permeability, with no off-target enhancement (Fig. 2). This parenchymal contrast enhancement resolved within 24 h after FUS treatment. MRI-based volumetric analysis of BBB opening in the hippocampus demonstrated parenchymal contrast enhancement of 95 ± 4% of the FUS targeted volume, ranging from 318 mm^3^ (two targets) to 873 mm^3^ (five targets). This BBB opening corresponds to 14 to 71% (average 29%) of the overall hippocampus volume given the variation in anatomy, hippocampal atrophy, dosing, and number of targets.

## Discussion

We demonstrate a safe, reproducible, and substantial BBB opening in six subjects with 17 distinct treatments targeting the deep and complex structure of the hippocampus. This study is the next step following a proof of concept phase I study in AD patients demonstrating safe BBB opening in a limited noneloquent white matter area of the superficial dorsolateral prefrontal cortex (11). The hippocampus and EC are complex definable deep brain structures, with interdigitating gray and white matter, significant vascularity, and proximity to the ventricle and skull base. Together, they are an important therapeutic target given the significant damage and atrophy in AD, their crucial role in memory and learning, and their involvement in the pathology of conditions like epilepsy and depression. In order to maximize the potential of targeted delivery of therapeutic agents, FUS-induced BBB opening must impact a sizeable volume of the target. We have demonstrated a reproducible pattern and substantial proportion of BBB opening (up to 71% of the overall hippocampal volume), which has not previously been shown in a specific structure of the human brain with FUS. Our results highlight the conformal capacity of FUS to safely and repeatedly open the BBB in the hippocampus, with spatial precision and no off-target BBB opening.

FUS-mediated BBB opening in preclinical models has been found to promote amyloid-beta clearance, delivery of endogenous antibodies, activation of microglia (8, 12), and potential modulation of the glymphatic system (13). The significance with respect to human translation of amyloid reduction observed in animal models with FUS-mediated BBB disruption is unknown. However, FUS provides a noninvasive method for time-limited, precise, and large BBB opening, providing an exciting opportunity for research coupling FUS with targeted delivery of medications, immunotherapy, gene therapy, or stem cells into various complex and deep brain structures, including the hippocampus.

In summary, we report an ongoing multicenter phase II trial demonstrating that a large volume of the BBB in the deep structure of the hippocampus and EC can be safely, reversibly, and repeatedly opened with spatial precision. The noninvasive, on-demand feature of FUS technology and focal BBB opening offers a unique opportunity for targeted delivery of therapeutics to meaningful volumes of essential brain structures in AD and other neurological conditions.

## Materials and Methods

This is an open-label, prospective phase II clinical trial sponsored by INSIGHTEC. It was designed to target the hippocampus and EC in patients with early AD for purposes of evaluating the safety, feasibility, and efficacy of repeated FUS-mediated BBB opening. The protocol was approved by the Food and Drug Administration and institutional review boards of West Virginia University and Weill Cornell Medical College. Participant eligibility criteria included the presence of early AD diagnosed with the National Institute of Aging – Alzheimer’s Association (NIA-AA) criteria (14), ^18^F-florbetaben PET scan consistent with AD and the presence of amyloid-beta plaques in the target, and no evidence of other CNS disease (ClinicalTrials.gov NCT03671889). Informed consent was obtained from all participants.

Participants underwent MR-guided, low-intensity FUS treatment at 220 kHz (ExAblate Neuro Type 2; INSIGHTEC) directed to the hippocampus/EC with simultaneous injection of i.v. microbubbles (Definity). The target hemisphere was selected based on individual anatomy, atrophy, pial/sulcal configuration, and presence of vessels, ependyma, and ventricles. Up to five 5 × 5 × 7 mm^3^ targets were selected based on hippocampal anatomy (Fig. 1 *C* and *D*). We started with a more conservative two to three targets in the first two participants and increased to four to five targets in participants 3 to 6 after safety was demonstrated. Targets were selected to maximally impact cortical and subcortical hippocampus and EC tissue while minimizing interfaces with vascular structures and ventricles. FUS treatment was administered in three sessions, separated by 2 wk, totaling 17 distinct treatments across six AD patients to date. Safety assessments, including neurological examination and T2* MRI to detect hemorrhage, were performed immediately after each treatment. Contrast-enhanced MRI with i.v. gadobutrol (0.1 mmol/kg) was performed immediately after FUS to evaluate BBB opening. The contrast-enhanced area was manually segmented by two neuroradiologists based on the gadolinium MRI T1 spoiled gradient recalled (SPGR) sequence and overlaid after linear image-based registration to the pre-FUS MRI (15). Contrast enhancement was compared to the FUS targeted volume and the overall hippocampal volume. Formal cognitive assessments were performed at the 30-d time point after final FUS treatment (*SI Appendix*). The pre- and post-FUS treatment MRI sequence as well necessary for the analysis are stored in GitHub (https://github.com/pd0033/PNAS-FUS.git) and will be made available upon acceptance of the paper to the readers.

## Supplementary Material

Supplementary File
